# Gastroprotective Effect of Geopropolis from *Melipona scutellaris* Is Dependent on Production of Nitric Oxide and Prostaglandin

**DOI:** 10.1155/2015/459846

**Published:** 2015-04-08

**Authors:** Jerônimo Aparecido Ribeiro-Junior, Marcelo Franchin, Miriam Elias Cavallini, Carina Denny, Severino Matias de Alencar, Masaharu Ikegaki, Pedro Luiz Rosalen

**Affiliations:** ^1^Department of Physiological Sciences, School of Dentistry of Piracicaba, University of Campinas, Avenue Limeira 901, 13414 903 Piracicaba, SP, Brazil; ^2^Department of Agro-Food Industry, Food and Nutrition, “Luiz de Queiroz” College of Agriculture, University of São Paulo, Avenue Pádua Dias 11, 13418-900 Piracicaba, SP, Brazil; ^3^Faculty of Pharmaceutical Sciences, Federal University of Alfenas, Rua Gabriel Monteiro da Silva 700, 37130-000 Alfenas, MG, Brazil

## Abstract

The aim of this study was to evaluate the gastroprotective activity of ethanolic extract of geopropolis (EEGP) from *Melipona scutellaris* and to investigate the possible mechanisms of action. The gastroprotective activity of the EEGP was evaluated using model ulcer induced by ethanol. To elucidate the possible mechanisms of action, we investigated the involvement of the nonprotein sulfhydryl (NP-SH) groups, nitric oxide and prostaglandins. In addition, the antisecretory activity of EEGP was also evaluated by pylorus ligated model. The EEGP orally administrated (300 mg/kg) reduced the ulcerative lesions induced by the ethanol (*P* < 0.05). Regarding the mechanism of action, the prior administration of nitric oxide and prostaglandins antagonists suppressed the activity of gastroprotective EEGP (*P* < 0.05). On the other hand the gastroprotective activity of EEGP was kept in the group pretreated with the antagonist of the NP-SH groups; furthermore the antisecretory activity was not significant (*P* > 0.05). These results support the alternative medicine use of geopropolis as gastroprotective and the activities observed show to be related to nitric oxide and prostaglandins production.

## 1. Introduction

Peptic ulcers are the imbalance between the aggressive agents (*Helicobacter pylori* and anti-inflammatory drugs, among others) and the protective agents (prostaglandins and nitric oxide, among others) [1, 2]. Despite the widespread use of different classes of monodrugs for the treatment of different types of ulcers, a large part of the world's population still benefits from the use of natural products [3].

The propolis is a nontoxic natural product, collected by bees from different plant parts [4]. Propolis has increased in popularity as an alternative medicine or dietary supplement, for improving health and preventing disease in various parts of the world [5]. Among the several biological activities of propolis reported in the literature antimicrobial, anti-inflammatory, anticancer, laxative, and antiulcer can be found [6–10].

The geopropolis, a mixture of resin, wax, and soil, is a propolis collected by a native stingless bee of the Meliponini tribe [11, 12] and also widely used in folk medicine for various therapeutic purposes [5].

The geopropolis from* Melipona scutellaris* bee species, popularly known as “uruçu” and found in northeastern Brazil, has been the focus of our research. Studies have shown that geopropolis has antinociceptive and anti-inflammatory properties, besides antimicrobial activity against different types of bacteria. In addition, studies on the chemical profile of geopropolis revealed absence of flavonoid and phenolic acids commonly found in propolis from* Apis mellifera* and presence of benzophenones [13–16].

Therefore, in order to aggregate scientific value to the geopropolis, this study aims at evaluating the gastroprotective activity of the ethanolic extract of geopropolis (EEGP) from* Melipona scutellaris* and at investigating the possible mechanisms of action.

## 2. Material and Methods

### 2.1. Obtaining the EEGP

The geopropolis samples were collected on municipality of Entre Rios (11°57′ S, 38°05′ W), state of Bahia, northeast of Brazil. The geopropolis samples (100 g) were extracted in 80% ethanol in water (w/v) at a 1/7 dilution rate at 70°C for 30 min, followed by filtration, thereby obtaining the EEGP. The same process was repeated twice. At the end, the EEGP was concentrated in a rotary evaporator at 40°C [13]. The EEGP dissolution was carried out in PBS 1 mM. The EEGP was administered to the animals by pathway oral (p.o.).

### 2.2. Animals

Male* Wistar* rats, SPF (specific-pathogen-free), weighing 200–250 g were provided by CEMIB/UNICAMP (Multidisciplinary Center for Biological Research, SP, Brazil) and kept in controlled temperature chambers (20 ± 2°C) in light-dark 12 hours cycles, relative humidity of 40 and 60%, with filtered water* ad libitum.* The animals fasted for 24 hours before the experiments. The procedures described were reviewed and approved by the local Animal Ethics Committee (CEUA Unicamp process number 2560-1).

### 2.3. Drugs and Reagents

The drugs were purchased from Sigma Chemical Co. St. Louis, MO, USA (N-ethylmaleimide, omeprazole, N*ω*-nitro-L-arginine methyl ester hydrochloride, and ethanol), MP Biomedicals (indomethacin), and Merck (organic solvents).

### 2.4. Gastric Lesion Induced by Ethanol

The rats were pretreated with EEGP at dose of 100, 200, or 300 mg/kg (p.o.). The positive control group received omeprazole 30 mg/kg (p.o.), and the negative control group received dissolving vehicle of EEGP (p.o.). One hour after the treatments, 1 mL of absolute ethanol was administered by p.o, and, one hour later, the animals were killed by anesthesia overload, and the stomach was removed and opened along the greater curvature [17]. The ulcerative lesions from each animal were calculated according to the Gamberini et al. [18] method, as described below.

### 2.5. Role of the Nonprotein Sulfhydryl (NP-SH) Groups on Gastroprotection Effect of the EEGP

The rats were pretreated with inhibitor of the NP-SH groups (N-ethylmaleimide 10 mg/kg, s.c.), 30 min prior to the treatment with EEGP (300 mg/kg) by p.o. [19]. The negative control group received dissolution vehicle of EEGP (p.o.). One hour after the treatments, 1 mL of absolute ethanol was administered by p.o., and, one hour later, the animals were killed by anesthesia overload, and the stomach was removed and opened along the greater curvature. The ulcerative lesions from each animal were calculated according to the Gamberini et al. [18] method.

### 2.6. Role of the Nitric Oxide on Gastroprotective Effect of the EEGP

The rats were pretreated with inhibitor from the nitric oxide synthase (L-NAME 5 mg/kg) by intraperitoneal (i.p.) administration, 30 min prior to the treatment with EEGP (300 mg/kg, p.o.). The negative control group received dissolution vehicle of EEGP (p.o.). One hour after the treatments, 1 mL of absolute ethanol was administered by p.o., and, one hour later, the animals were killed by anesthesia overload, and the stomach was removed and opened along the greater curvature [19]. The ulcerative lesions from each animal were calculated according to the Gamberini et al. [18] method.

### 2.7. Role of Prostaglandins on Gastroprotective Effect of the EEGP

The rats were pretreated with the cyclooxygenase inhibitor (indomethacin 5 mg/kg, i.p.), 30 min before the EEGP treatment (300 mg/kg, p.o.). The negative control group received dissolution vehicle of EEGP (p.o.). One hour after the treatments, 1 mL of absolute ethanol was administered by p.o., and, one hour later, the animals were killed by anesthesia overload, and the stomach was removed and opened along the greater curvature [19]. The ulcerative lesions from each animal were calculated according to the Gamberini et al. [18] method.

### 2.8. Evaluation of the Antisecretory Activity

The animals were anesthetized, their abdomen was dissected, and the pylorus was connected. Immediately after this procedure, the EEGP 300 mg/kg, cimetidine 100 mg/kg, or dissolution vehicle of EEGP (negative control) was administered by p.o. to the respective group of animals. The abdomens were sutured and, four hours later, the animals were killed. The stomachs were removed and the gastric content was collected and centrifuged. The volume and the pH of the gastric juice were measured [20].

### 2.9. Statistical Analysis

Data were expressed as means ± standard error of the mean (SEM) and statistical comparison between groups was made utilizing analysis of variance (ANOVA) followed by test of Dunnett or Tukey. Significance was accepted when *P* < 0.05.

## 3. Results and Discussion

This study evaluated the gastroprotective activity of EEGP from* Melipona scutellaris*, as well as the possible action mechanisms involved.

The geopropolis used in this work presents the same chemical composition as described in our previous publications, where absence of flavonoids and phenolic acids and presence of benzophenone were found [15, 16].

**Figure 1:**
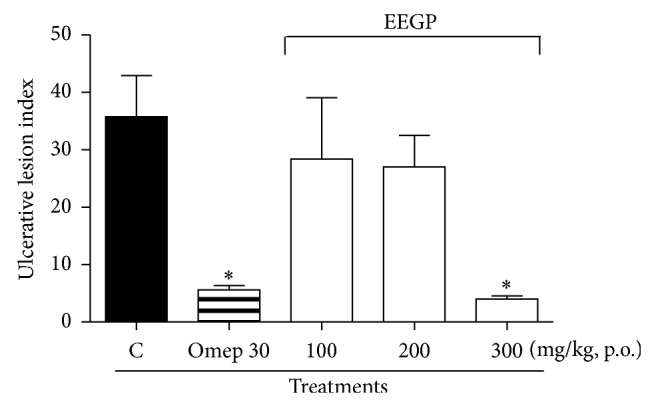
Effect of the p.o. administration of ethanolic extract of geopropolis (EEGP) on the ethanol induced ulcers. Control (C) treated with vehicle, omeprazole 30 mg/kg (Omep 30), and EEGP with doses of 100, 200, and 300 mg/kg. The results are expressed as means ± SEM, *n* = 5. Symbols indicate statistical difference (ANOVA followed by Dunnett test, *P* < 0.05). ∗ compared to the control group.

Ethanol is a potent gastric ulcer inducer agent, and it is widely applied to evaluate the gastroprotective activity of plant extracts, as well as new pharmaceutical drugs in animal models. Its effect causes an imbalance between the oxidizing and the antioxidants agents in the gastric mucosa. This imbalance causes bleeding resulting from the ruptured blood vessels [21, 22]. In the present study, we evaluated the EEGP gastroprotective activity on the ethanol-induced ulcer model (Figure 1). The EEGP (300 mg/kg) orally administrated decreased the ulcerative lesions, where an 89% reduction was detected compared to the negative control group (*P* < 0.05). Regarding the positive control group (omeprazole), there was an 85% reduction of the ulcerative lesions compared to the negative control group (*P* < 0.05).

To elucidate the possible mechanisms of action involved with the gastroprotective activity of EEGP, we investigated the participation of the NP-SH groups, nitric oxide and prostaglandins. In addition, the antisecretory activity of EEGP was also evaluated.

**Figure 2:**
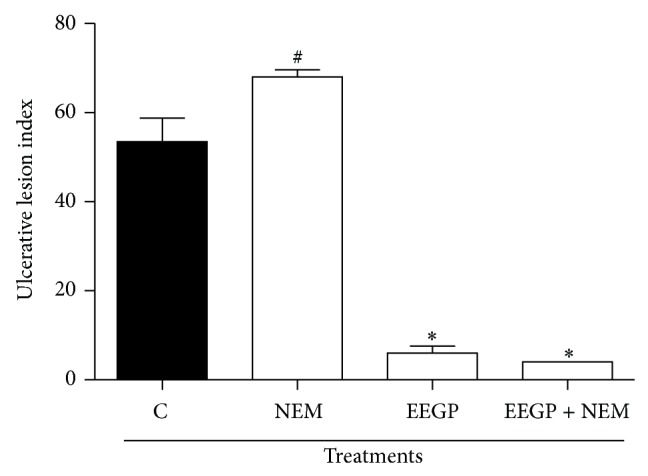
Effect of the p.o. administration of ethanolic extract of geopropolis (EEGP) on the ethanol induced ulcers. Rats pretreated subcutaneous (s.c.) with the N-ethylmaleimide 10 mg/kg (NEM). After 30 min, the rats were treated with vehicle (C and NEM) and with 300 mg/kg doses of EEGP. The results are expressed as means ± SEM, *n* = 5. Symbols indicate statistical difference (ANOVA followed by Tukey test, *P* < 0.05). # compared to control (C) group and ∗ compared to control group.

The NP-SH groups are protective of the gastric mucosa. In this case, the role of these mediators is associated with the free radicals blockage in the gastric mucosa [23]. Thus, in order to evaluate the EEGP association with these substances, the animals underwent a pretreatment with a blocker agent (N-ethylmaleimide 10 mg/kg) of the NP-SH groups. The results showed that the administration of the N-ethylmaleimide (Figure 2) did not suppress the gastroprotective activity of the EEGP (*P* > 0.05). Thus, suggesting that the EEGP activity is not associated with NP-SH groups.

**Figure 3:**
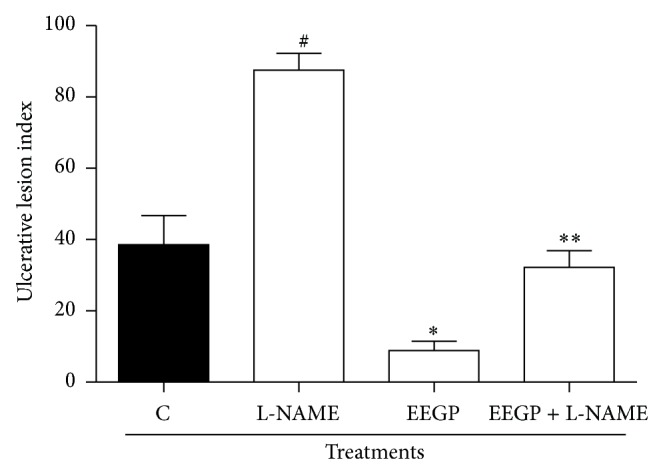
Effect of the p.o. administration of ethanolic extract of geopropolis (EEGP) on the ethanol induced ulcers. Rats pretreated with 5 mg/kg of the L-NAME (i.p.). After 30 min, the rats were treated with vehicle (C and L-NAME) and 300 mg/kg doses of EEGP. The results are expressed as means ± SEM, *n* = 5. Symbols indicate statistical difference (ANOVA followed by Tukey test, *P* < 0.05). # compared to control group; ∗ compared to control (C) group; ∗∗ compared to EEGP group.

Nitric oxide plays a vital role in gastric gastroprotection also. Among its actions are regulation of gastric secretion and gastric stimulation of mucus secretion [24, 25]. Furthermore, studies have shown that NO is able to decrease the adhesion of neutrophils to endothelial cells during the inflammatory process [26]. The pretreatment with L-NAME (inhibitor of synthesis of nitric oxide) suppressed the gastroprotective activity of EEGP (Figure 3, *P* < 0.05). These results corroborate the study of Franchin et al. [14], where it was observed that the administration of inhibitors of nitric oxide production suppressed the anti-inflammatory activity of the EEGP. In the same study, it was found that the activity of EEGP on the inflammatory process was related to the increased production of nitric oxide (verified by quantifying nitrite), which resulted in decreased adhesion of neutrophils on endothelial cells. Therefore, the EEGP increased the gastroprotective response and reduced the ulcerogenic effect induced by ethanol in the gastric mucosa, probably by increased production of NO.

**Figure 4:**
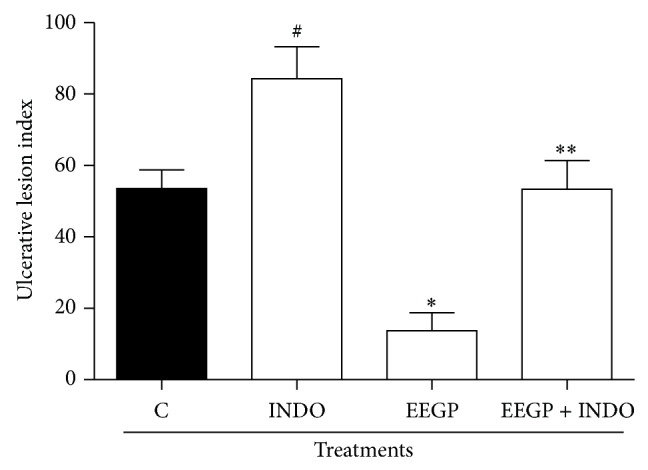
Effect of the p.o. administration of ethanolic extract of geopropolis (EEGP) on the ethanol induced ulcers. Rats pretreated with 5 mg/kg of the indomethacin-INDO (i.p.). After 30 min, the rats were treated with vehicle (C and Indo) and 300 mg/kg doses of EEGP. The results are expressed as means ± SEM, *n* = 5. Symbols indicate statistical difference (ANOVA followed by Tukey test, *P* < 0.05). # compared to control group; ∗ compared to control (C) group; ∗∗ compared to EEGP group.

This study also evaluated the role of prostaglandins in the EEGP gastroprotective activity. Indomethacin, a nonsteroidal anti-inflammatory drug, is widely used for induction of ulcers in animal research. These drugs are known to inhibit the production of prostaglandins, including those that have protective action in the gastric tissue [27, 28]. The prostaglandins produced in the stomach exert gastroprotective activity through the stimulatory action of the gastric mucus and of bicarbonate secretion [29]. According to the results (Figure 4), it was found that the prior administration of indomethacin in ulcer model induced by ethanol reverted the EEGP gastroprotective activity (*P* < 0.05). These results suggest that the EEGP beyond develops its gastroprotective activity through modulation of nitric oxide and also acts by increasing the levels of prostaglandins present in the gastric mucosa.

Finally, we evaluated the antisecretory activity of the EEGP. Acetylcholine, histamine, and gastrin are endogenous substances responsible for the regulation of acid secretion [30]. Currently, antiulcer drugs act by blocking the acid secretion, for example, omeprazole (proton pump blockers) and cimetidine (H_2_ inhibitor) [31].

The model of ligature of the pylorus of the stomach in mice is widely used to study the antisecretory activity of new drugs. This fact is understandable due to the effect generated by ligation of the pylorus, which is the acids hypersecretion on stomach [20]. According to Table 1, it is observed that the volume and pH of gastric juice remained unaltered in the stomachs of animals submitted to binding the pylorus remained (*P* > 0.05), after administration of EEGP (300 mg/kg). On the other hand the positive control (cimetidine 100 mg/kg) not only decreased the gastric juice volume, but also increased the pH (*P* < 0.05). These results, therefore, suggest that the gastroprotective activity of the EEGP is not related to the regulation of gastric secretion.

## 4. Conclusion

The results of this study indicate that geopropolis exerts a gastroprotective effect in rats with ethanol-induced gastric mucosal damage. The results also suggest that the gastroprotective effect of geopropolis could be related to the gastroprotective mechanism in which nitric oxide and prostaglandins are involved.

## Figures and Tables

**Table 1:** Evaluation of the anti-secretory activity of the ethanolic extract of geopropolis (EEGP) using the pyloric ligation model. Means ± SEM, *n* = 5.

Treatments	Dose (mg/kg)	Volume (mL)	pH
Control	—	3.3 ± 0.6	3.4 ± 0.5
Cimetidine	100	1.9 ± 0.3^∗^	6.4 ± 0.1^∗^
EEGP	300	2.6 ± 0.5	3.4 ± 0.5

^∗^Compared to control group (ANOVA followed by Dunnett, *P* < 0.05).

## Abbreviations

EEGP: Ethanolic extract of geopropolis
Indo: Indomethacin
NEM: N-Ethylmaleimide
NP-SH: Nonprotein sulfhydryl
OMEP: Omeprazole
L-NAME: N*ω*-nitro-L-arginine methyl ester hydrochloride.
